# The COVID‐19 pandemic and young people's civic engagement: A scoping review

**DOI:** 10.1111/jora.13039

**Published:** 2024-12-01

**Authors:** Ingrid Schoon, Shanu Shukla, Suman Verma, Eden Terol, Josafá Moreira Da Cunha

**Affiliations:** ^1^ Social Research Institute University College London London UK; ^2^ Interdisciplinary Research Team on Internet & Society, Faculty of Social Studies Masaryk University Brno Czech Republic; ^3^ Panjab University Punjab India; ^4^ Applied Psychology Program University of the Philippines, Diliman Extension Program in Pampanga Pampanga Philippines; ^5^ School of Education Federal University of Parana Parana Brazil

**Keywords:** civic engagement, COVID‐19, positive youth development, scoping review

## Abstract

This scoping review summarizes evidence regarding the impact of civic and community engagement of young people during the COVID‐19 pandemic. Recognizing that the global pandemic not only brought challenges but also new opportunities to take a stance and to actively engage in communities and society, this review assesses the impact of the COVID‐19 pandemic on young people's civic engagement across different cultural contexts and identifies key factors and processes that enable young people to engage with their community or society at large. We summarize evidence from 27 original research papers, one thought piece, and four reports conducted by global organizations such as the United Nations and OECD. Relevant research was conducted in the United States, Europe, China, Southeast Asia, South Africa, and New Zealand, addressing the development of leadership skills, civic responsibility, critical consciousness, civic and community engagement, as well as social integration. Key factors that facilitated civic engagement include national investments in online learning facilities, support for basic needs (such as education, health, and employment), and promotion and encouragement of local initiatives. The studies differed in their focus depending on the socio‐cultural context encountered and future research needs to consider cultural variations and different demands on young people to inform effective practices for supporting young people's active engagement in society.

## INTRODUCTION

The COVID‐19 pandemic affected every dimension of young people's lives, impacting their education and training (Chaturvedi et al., 2021; Engzell et al., 2021; Green et al., 2022), their employment prospects (Hlasny & AlAzzawi, 2022; Major & Machin, 2020; Rotar, 2022), their outlook to the future (Arslan & Yıldırım, 2021; Gadermann et al., 2022; Hussong et al., 2021; Schoon & Henseke, 2022), as well as their mental health (Nearchou et al., 2020; Paterson et al., 2021; Racine et al., 2021; Samji et al., 2022). However, the pandemic also opened up new opportunities for civic and community engagement (Kwan, 2022a; Pavarini et al., 2020; United Nations, 2021). Active youth engagement can be understood as an indicator of positive youth development (PYD), suggesting a sense of an interdependent self, cultivating basic skills and capacities such as empathy, respect for others, responsibility, resilience, prosocial, and cooperative behaviors (Lerner et al., 2003; Sherrod, 2015). These capacities, in turn, can be vital to cope with the challenges of a global pandemic. A UNICEF report defines civic engagement as “individual or collective actions in which people participate to improve the well‐being of communities or society in general” (Cho et al., 2020, p. 6). Everywhere, and every day, young people are acting on the issues that affect them, including the climate crisis, conflicts and war, disasters, gender inequalities, political exclusion, governance failures, or lack of quality education, health, and decent jobs for youth. There is evidence to suggest that before the pandemic globally one in three young people were active volunteers (nearly 600 million youth worldwide) and that youth online volunteering has been a key resource during the COVID‐19 pandemic (UN Volunteers‐UNDP, 2021). Other forms of civic participation during the pandemic included community support initiatives, such as organizing and distributing food to those in need, providing virtual tutoring or mentorship, and participating in digital campaigns to raise awareness about pandemic‐related issues (United Nations, 2022).

The aims of this scoping review are to (1) review the existing evidence on the impact of the COVID‐19 pandemic on young people's civic engagement; (2) assess manifestations of civic engagement across different cultural contexts; and (3) identify key factors and processes that enable young people to engage with their community or society at large during the global COVID‐19 pandemic. While most previous research focused on the effects of COVID‐19 on young people's education, employment, health, and well‐being, there has been less concern about their engagement in society. Moreover, much of the research has focused on the experiences of young people in more economically developed or “WEIRD” (Western, Educated, Industrialized, Rich, and Democratic) countries, with less evidence from low‐to‐middle‐income countries. Considering research published in both more or less economically developed countries, this review aims to identify important knowledge gaps and differences in the approach and conceptualization of active youth civic engagement.

### Civic engagement as an expression of positive youth development

Youth civic engagement is understood as a multidimensional concept characterized by individual or collective actions that demonstrate a commitment to improving the well‐being of the community and society at large (Cho et al., 2020; Lerner, 2004). This can comprise political and prosocial contributions to society, including voting, political engagement, informal helping, and volunteering (Ekman & Amnå, 2012; Miller et al., 2021; Shaw et al., 2014; Youniss et al., 2002). Moreover, youth civic engagement is associated with processes of identity formation, health, and well‐being, as well as cognitive and emotional development, for example as a way of coping with macro‐level stressors like the pandemic (Wray‐Lake & Ballard, 2023).

PYD approaches emphasize the strengths and potential of youth and argue that development can be supported by individual and contextual resources (Lerner et al., 2021). Understanding civic engagement as an expression of PYD enables the assessment of the conditions that enable civic engagement and the bi‐directional transactions between individual and context, that is, how external resources, such as community assets for instance, can promote youth engagement, and how youth engagement, in turn, contributes to the building of community assets (Sherrod, 2007, 2015). Moreover, such an approach is open to the consideration of the positive potential that might arise out of challenging circumstances (Flanagan, 2013; Youniss et al., 2002), such as the COVID‐19 pandemic. Young people make up almost a third of the world's population and are the world's most important asset, being catalysts of inclusive and resilient societies in crisis response, recovery, and preparation for future shocks (OECD, 2020). Providing effective support for their civic development is vital since enabling them to develop positively will give them the power to support themselves, their families, their communities, and society at large (Hempel et al., 2012; Lerner et al., 2003; Petersen et al., 2016).

There are a number of established models and approaches conceptualizing PYD, all of which originated from socio‐ecological developmental system theories (Bronfenbrenner & Ceci, 1994) emphasizing the reciprocal interactions between a developing individual and a changing social context. PYD models specify the developmental assets that facilitate positive development (Benson et al., 2011; Catalano et al., 2004; Lerner et al., 2005, 2010, 2021), also considering cultural variations in relevant influences (Catalano et al., 2019; Shek et al., 2007). For example, Benson and colleagues proposed the 40 Developmental Assets Framework, differentiating between 20 internal (i.e., personal skills or character strengths) and 20 external (i.e., social relationships, empowerment, and opportunities created by the family, school, community, or peers) assets that support PYD (Benson et al., 2011). In addition, social justice principles and critical consciousness are considered to play a crucial role in PYD recognizing the embeddedness of individual lives in systems of privilege and oppression and the need to enable thriving among marginalized youth (Gonzalez et al., 2020; Sherrod et al., 2005; Shields, 2010; Tyler et al., 2020). Critical consciousness refers to the process through which marginalized individuals engage in critical reflection on their social conditions and become motivated to engage individually or collectively in actions to challenge social inequities and oppression (Diemer et al., 2016; Freire, 1978). Indeed, it has been argued that critical reflection plays a pivotal role in encouraging youth to understand questions of societal inequalities, political efficacy, and their belief in their ability to affect change, empowering individuals to become active agents of change, thereby promoting both personal growth and social justice.

Despite differences in defining PYD across cultures, different models share key similarities in particular regarding their focus on (a) the strengths of young people, (b) the plasticity of human development; and (c) the inter‐linkages of internal assets (such as individual competencies) and external resources (such as community support) to support PYD (Catalano et al., 2019; Lerner et al., 2021). Understood within a socio‐ecological PYD approach youth civic engagement is a crucial example of the reciprocal relationship between individuals, their communities, culture, and societies: of how the wider context can facilitate civic engagement of young people and how youth's civic involvement can contribute to the building and maintaining of civil society. Existing evidence from studies conducted in low‐to‐middle‐income countries highlights the role of contextual influences for PYD, in particular the benefits of providing opportunities for young people to engage in meaningful ways with their communities and to strengthen the social fabric of their lives (Petersen et al., 2016).

### Civic engagement during the COVID‐19 pandemic

The outbreak of the COVID‐19 pandemic reshaped the public sphere and opportunities for citizen's participation. Across the globe, the pandemic forced countries to adopt lockdown measures and restrictions that prevented physical contact and social gatherings and significantly shifted the way people interact and maintain social connections. Although there were country‐specific differences in response based on their different economic, cultural, and health system situations, there has generally been increasing use of digital technology to stay in touch with friends, family, and colleagues (Kwan, 2022b; Wilf et al., 2023). In addition, new areas of youth engagement have emerged (OECD, 2020; United Nations, 2021; United Nations Development Programme, 2021) involving both offline and online activities (e.g., shopping and delivery services for people who need help, volunteering, participation in charity events, or political engagement) aimed at addressing the social, medical, and economic emergency situation. Yet, despite the global appeal that “we are all in this together,” there were persisting inequalities regarding exposure to risks associated with access to education, health, employment, and housing, undermining individual capacities to cope (Settersten et al., 2020; United Nations, 2022). Indeed, the pandemic exacerbated preexisting social inequalities, including the experience of poverty (OECD, 2020), gender and racial discrimination, access to vaccination and health care (Sachs et al., 2022), as well as inequalities in access to digital resources (Beaunoyer et al., 2020) – a vital source for many to stay socially engaged. Although the immediate health threats of the pandemic associated with a new virus have now receded, we cannot be complacent in sight of possible future global crises, health threats, threats to our environment, increasing social inequalities and polarization, economic upheavals, and shortages of supplies.

In these times of growing uncertainty, we must also rethink pathways to nurture civic engagement, which is critical to addressing compounding societal challenges. The COVID‐19 pandemic has led to a global breakdown of support systems and societal promises—echoing the uncertainty faced by many youths in Eastern Europe after the fall of the Soviet Union, who were left questioning whether society cared for them and whether they should actively engage with a society that had broken its promises (Flanagan, 2013). The uncertainty and economic upheaval that followed the COVID‐19 pandemic hit young people hard, particularly those from marginalized backgrounds (OECD, 2020), who are bearing the brunt of this transition into a new, less defined/stable social contract. Previous evidence from the aftermath of the 2008 Great Recession suggests that in a context of instability and uncertainty trust in institutions has declined, while support and concern for others have increased (Schoon & Mortimer, 2017). Examining the factors and processes that support the civic engagement of young people during a global crisis can thus provide critical insights into how to prepare society for future emergencies.

### The present study

Against this background a scoping review was conducted, summarizing evidence from studies assessing the impact of the COVID‐19 pandemic on the civic engagement of young people across diverse cultural contexts. The focus of the review is on young people aged 16–30 which reflects a critical period in the transition from adolescence to adulthood. In particular, the later school years and early adulthood are crucial times for defining one's role as a member of society (Erikson, 1968). Due to changing education and employment opportunities across many countries, the transition to adulthood has extended into the late 20th and early 30s, a period also described as emerging adulthood (Arnett, 2014). We thus use a relatively wide age range to be inclusive of cultural variations in the timing of this transition period. We ask (1) what was the impact of the COVID‐19 pandemic on young people's civic engagement; (2) which dimensions of civic engagement have been addressed across different cultural contexts; and (3) what are the key factors and processes that enabled young people to engage with their community or society at large during the global COVID‐19 pandemic. In our review, we include quantitative, qualitative as well as mixed‐method studies conducted in different cultural contexts, published between January 2020 and January 2023 to capture experiences during the onset and most critical phase of the pandemic. As such, this scoping review aims to provide a better understanding of how COVID‐19 impacted young people's civic engagement, expose gaps in the current evidence base, assess quality, and provide researchers, practitioners, and policymakers with an overview of key outcomes and future directions.

## METHODOLOGY

### Data source and search strategy

The literature search focused on studies published between January 2020 and January 2023 and was independently conducted by two of the co‐authors drawing on five electronic databases: PubMed, Web of Science, Scopus, EBSCO, and the first 200 results from Google Scholar. The databases chosen for this review were selected to ensure comprehensiveness and coverage across a wide range of disciplines. Reference lists of the retrieved articles were also scanned. The search terms focused on: covid OR coronavirus OR corona OR “COVID‐19” OR pandemic AND “civic engagement” OR empowerment OR “youth participation” AND “positive youth development” OR PYD. For details of search terms used in specific databases refer to Table S1. In addition, we conducted a web search on Google (www.google.com) to identify gray literature, focusing on reports by established organizations.

### Eligibility criteria and study selection

A three‐stage screening process was used to assess the relevance of studies identified in the search for inclusion in the review. For the first level of screening, only the title and abstracts were reviewed. The second level of screening comprised a review of the full texts, and the third level involved a quality assessment of the articles described in more detail below. Articles were rejected if they did not meet the five inclusion criteria (a) focus on youth civic engagement and PYD during the COVID‐19 pandemic; (b) age range of 16–30 years; (c) publication between January 2020 to January 2023; (d) qualitative, quantitative, or mixed‐method studies; and (e) written in English. Regarding our selection of reports, we focused on reports from global organizations such as the United Nations and OECD.

### Quality assessment

The quality assessment was conducted by the 5 authors (the reviewers). To gauge the quality of included empirical studies, we used the 16‐item Quality Assessment Tool for Studies with Diverse Designs (QATSDD) tool (Sirriyeh et al., 2012). This appraisal tool comprised 16 items (14 items for qualitative/quantitative studies; 16 for mixed method) that are scored using a four‐point scale (0–3), with a maximum possible of 42 points (qualitative/quantitative studies) and 48 points (mixed methods). The five reviewers were grouped in multiple pairs and independently scored the selected papers. A total of 25 empirical articles (quantitative = 12; qualitative = 9; and mixed method = 4) underwent quality assessment (no quality assessment was done for a thought piece (*n* = 1), a case study (*n* = 1), a thesis (*n* = 1), conference proceedings (*n* = 1), and the reports (*n* = 4)).

The scoring ranged from 6 to 44 points, indicating the mixed quality of the selected papers. We excluded studies that did not reach a minimum score of 14. The inter‐rater reliability was calculated using a weighted Cohen's kappa (Cohen, 1968) and yielded a score in the range of 0.80 to 1.

### Data charting

Data analysis began with the extraction of the following data: authors' information, year of publication, sample size, sample characteristics (age, gender, and country), study design, research type and focus (i.e., which aspects of civic engagement), as well as PYD perspectives mentioned. The charted data from primary research reports formed the basis for further analysis.

### Collating, summarizing, and reporting the results

The retrieved data were analyzed using thematic analysis (Thomas & Harden, 2008). First, a preliminary synthesis of the findings of the included studies was developed. Secondly, the five reviewers explored varying characteristics between studies, while also grouping findings considered conceptually similar into “descriptive themes.” The reviewers were divided into multiple pairs to control for potential coding bias. Themes and subthemes were initially generated inductively from the raw data, and then reviewed and vetted by the reviewers by returning to the actual studies to establish referential adequacy. In our regular online team meetings, we discussed whether the themes were specific enough and broad enough to clearly and succinctly capture the set of themes contained in the studies and reports. The review process was iterative and continued until the final set of themes was agreed upon by all reviewers.

## RESULTS

**FIGURE 1:**
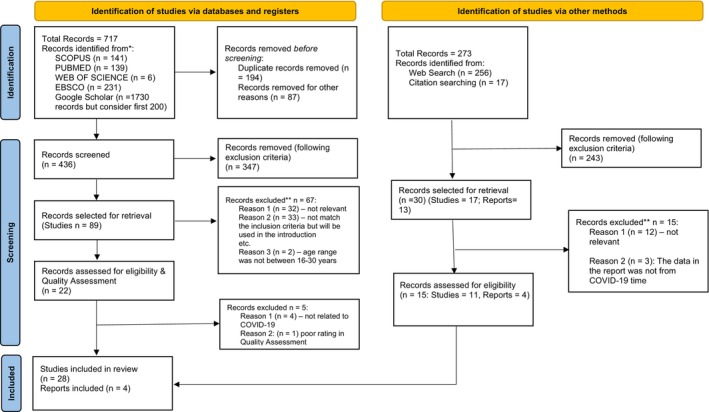
PRISMA flow chart. *Reporting the number of records identified from each database or register searched. **All records excluded by a human.

As shown in the PRISMA flow diagram (Figure 1), our literature search first identified 717 records through five databases (Scopus, PubMed, Web of Science, EBSCO, and Google Scholar). After removing duplicates and other exclusion criteria, 436 records were screened. The full texts of these records were retrieved. After careful examination, 347 records were removed. Eighty‐nine records were sought for retrieval and distributed among the four co‐authors for screening (joint task conducted in pairs). The authors met throughout the screening process to resolve conflicts and discuss any uncertainties related to study selection (Levac et al., 2010). After reading the full text, a further 67 records were excluded, with the overall kappa for the selection ranging from .97 to 1, where a kappa of greater than 0.8 is considered to represent a high level of agreement. In the final stage of screening, 22 records were again assessed for eligibility and quality assessment, and finally, 17 were selected for the scoping review.

Similarly, we identified a further 273 records through web searching (via Google) and citation searching. After the removal of duplicates and applying the exclusion criteria, 30 records (17 studies and 13 reports) were selected for retrieval. After reading the complete text, 15 records were excluded, and 15 were further assessed for eligibility and quality assessment. Throughout the screening process, study selection and identification of records were done through mutual agreement. Finally, 15 records (11 studies and four reports) were selected for the scoping review. Thus, the scoping review comprises 28 studies and four reports from two identification processes. The rate of agreement was nearly 100%. The reports included studies commissioned by the Organization for Economic Cooperation and Development (OECD), the United Nations (UN), and the United Nations Development Program (UNDP).

Table 1 provides an overview of all records included in the scoping review. The studies comprised quantitative studies (*n* = 13), qualitative (*n* = 10), mixed‐method studies (*n* = 4), and a thought piece (*n* = 1). The research articles stem from the United States (*n* = 9), China (*n* = 7), Europe (*n* = 6), Southeast Asia (*n* = 3), South Africa (*n* = 2), and New Zealand (*n* = 1) drawing on different theoretical frameworks. A number of studies adopted a multi‐dimensional PYD approach as informed by socio‐ecological systems models (Benson et al, 2011; Catalano et al., 2005; Lerner et al., 2005). Another study by Wati et al. (2021), as well as the four reports did not explicitly mention PYD theories but implemented a youth empowerment and human rights perspective. Some studies drew on the notion of critical consciousness (Fine et al., 2021; Maker Castro et al., 2022; Wilf & Wray‐Lake, 2021), as well as transformative social and emotional learning (Owens et al., 2022).

**TABLE 1:** Overview of the reviewed studies.

SNo.	Study	Type	*n*	Focus	Sample	Period	Location	Positive youth development (PYD) perspective	Key findings
1.	Arnold (2020)	Thought piece	–	PYD	–	–	USA	Developmental Assets (Benson, Lerner)	The article argues for public investment in Positive Youth Development and out‐of‐school programs. It stresses the importance of giving a voice to young people and supporting them in facing challenges such as mental health issues, educational disruptions, and unemployment. Additionally, it calls for enhancing civic discourse and engagement among young people
2.	Branquinho et al. (2022)	Mixed method (Case Study)	*n* = 437 Quantitative *n* = 32, Qualitative	Active citizenship, social participation, social entrepreneurship	14–30 years *M*_age_ = 18.36 years (Quantitative) *M*_age_ = 19.43 years (Qualitative)	May 2020–August 2020	Cascais, Portugal	Lerner PYD; active citizenship, social participation, social entrepreneurship	The study investigated the impact of COVID‐19 on young people's civic engagement and positive youth development (PYD) in Cascais, Portugal, including a survey and a case study evaluating a civic engagement initiative. Overall, participants showed a good sense of belonging and social self‐efficacy, with girls exhibiting higher community participation expectations and active participation, while boys exhibited higher levels of competence. The case study revealed higher engagement in issues related to substance use, social capital, and sexuality. Participants perceived increases in their action‐research skills and knowledge, as well as in civic engagement. The results highlight the need to encourage civic engagement among youth by enhancing both their knowledge and skills for action
3.	Castro et al. (2022)	Quantitative	*n* = 707	Critical consciousness and its association with well‐being among marginalized youth enrolled in college	18–22 years (*M*_age_ = 20 years)	Late April 2020	Across 49 USA states and Washington DC	Critical consciousness	This survey of US college students shows that critical reflection and action were associated with anxiety for the full sample with no evidence for moderation by socio‐demographic factors. Among LGBT+ youth critical reflection was associated with hopefulness. The findings suggest the need for a more differentiated view of how critical consciousness is associated with well‐being among distinct marginalized groups
4.	Chai et al. (2022)	Quantitative (pretest–posttest design)	*n* = 630	e‐service learning Leadership	16–20+ years	2021–2022	Hong Kong	Catalano 15 PYD construct—Chinese version	This study assessed the effectiveness of a hybrid‐mode leadership course based on PYD for university students during COVID‐19. It employed a pretest and posttest design (*n* = 630) to measure changes in PYD, well‐being, and desired graduate competencies (e.g., critical thinking). The results showed significant improvement in PYD indicators and overall well‐being, with high student satisfaction regarding the course design and teaching. Moreover, students' satisfaction with the curriculum positively predicted their improvement in indicators of PYD
5.	Chauke (2020)	Qualitative/thematic Analysis	*n* = 10	Civic engagement PYD	20–35 years Purposive Sampling	–	Cape Town, South Africa	Maslow hierarchy of needs; Developmental assets (Benson); resourcefulness of young people	This study focused on how young people in Cape Town (South Africa) actively engaged in the combat of COVID‐19. Using a qualitative approach, it explores how young people have adapted to the pandemic by engaging in digital initiatives, community screening, distribution of food parcels, social distancing enforcement, and educational campaigns. These efforts highlight the youth's adaptability and resilience, demonstrating a shift toward digital platforms for information dissemination and community support. The study emphasizes the importance of youth‐led projects in enhancing community engagement and resilience during crises, suggesting that young people's proactive involvement in various initiatives has positively impacted their communities and personal development
6.	Chauke (2022)	Qualitative/Thematic Analysis	*n* = 20	Engagement with the National Youth Service Program during the Covid‐19 pandemic	18–35 years Convenience Sampling	–	Cape Town, South Africa	Ladder of participation theory	This qualitative study uses interviews to explore engagement with the National Youth Service Program in Cape Town. Findings show how insufficient support during lockdowns, such as not addressing youth unemployment and poverty, eroded trust in the government, leading to political disengagement. The conclusion highlights how socio‐economic challenges, and not disinterest, are critical obstacles to youth voting and civic engagement
7.	Fine et al. (2021)	Qualitative/Oral history	12 youth researchers collected and analyzed 48 interviews	Critical participatory action research guided by a commitment to decolonizing methodologies and inclusive, evidence‐based activism	14–16 years	–	USA, Washington	Critical consciousness; race theory and radical hope	This paper draws on a five‐year youth oral history project involving immigrant youth of color and educators. Initially focused on documenting generational experiences of inequities, policing, housing, and immigration struggles, the study later investigated the impact of COVID‐19, uprisings, and remote learning on youth of color. Key findings emphasize the transformative potential of culturally responsive education in fostering critical consciousness and civic engagement, advocating for anti‐racist developmental science
8.	Ingoglia et al. (2022)	Quantitative	*n* = 2873	Responsibility toward community Alignment of personal and community interests assessed through the appraisal of public health emergency management	19–30 years (*M*_age_ = 22.67 years)	March 2020 to April 2020	Italy	Civic competence	This study explored how personal attributes (civic competence, interdependent self‐construal) and community resources (sense of community, civic engagement) among 2873 Italian emerging adults impacted their perception of COVID‐19 public health policies and efforts to prevent contagion. Results indicated that higher levels of civic competence directly foster a stronger sense of community, which then positively impacts attitudes toward public health measures. Additionally, this sense of community indirectly enhances proactive prevention behaviors
9.	Jiang and Gu (2022)	Qualitative/Content analysis (video documentation of civic participation)	2 videos	Digital civic engagement during the Covid‐19 pandemic	No ages given; videos produced by two undergraduate degree students	videos uploaded on 2 February 2020— (the start of the pandemic)	China	Participatory culture and digital citizenship	This study delves into digital multimodal composing (DMC) through a multimodal analysis of COVID‐19‐related videos within a virtual ethnography of Chinese social media. The study explores how video‐makers utilized multimodal techniques for civic participation during a crisis. It illustrates two new modes of digital civic engagement involving popularizing scientific knowledge of safe living during the COVID‐19 pandemic and inspiring people to fight the virus with courage and hope. The study advocates DMC as a powerful channel for civic involvement, discussing implications for civic purposes and digital citizenship education
10.	Kvieskienė et al. (2021)	Mixed‐method (quantitative and review of local initiatives and country‐specific case studies)	–	Social policies to address rural NEET populations in Baltic countries	15–29 years	–	Rural areas of Baltic States	Social integration	Reviews evidence from municipal and local government initiatives to address the risk of social exclusion among rural NEET (not in Education, Employment, or Training) populations who face particular challenges such as lack of access to second chances, apprentice, or mentoring programs, especially during the pandemic. Effective measures aiming to support integration did raise self‐confidence, address mental health problems, and promote mobile work, networking, development of skills, and creativity. The study highlights the need for integrated services, flexible and sustainable programs (prolonged mentoring) that are locally planned and implemented with the aim to meet individualized needs. Yet lack of good data prevents more detailed analysis
11.	Kwan (2022a)	Qualitative/thematic analysis	Thematic analysis of 17 podcasts with 20 individual	Political awareness and activism, voting, instigating political change	19–28 years (*M*_age_ = 24.4 years) Purposive sampling	June/July 2020	Singapore	Political participation, Civic engagement	Using public podcast episodes produced and published by the author during Singapore's COVID‐19 lockdown, this exploratory study seeks to first describe the motivations for and processes of civic and community engagement in the immediate aftermath of COVID‐19. Next, it aims to understand how engagement during the pandemic shaped youth commitments to future action and the potential directions of their future socio‐political engagement with communities and governments The findings suggest that civic engagement during the pandemic impacted community‐building and young people's expectations of their governments
12.	Kwan (2022b)	Qualitative/thematic analysis	Thematic analysis of 15 podcast episodes with 30 individual	Revealed five chronologically related themes: “Pandemic and lockdown as triggers,” “Motivations,” “Online mobilization,” “Action,” and “Future directions.	20–31 years (*M*_age_ = 23.8 years)	April/June 2020	Singapore	Civic and community engagement	Results from the study show that students with higher levels of psychological well‐being have higher levels of civic engagement. Yet, the overall level of civic engagement of academic students is low. Volunteering and donation were the forms of civic engagement that students participating in our research used less often. 6 out of 10 students have not participated in any volunteering activities during the 12‐month COVID‐19 outbreak
13.	Leung et al. (2021)	Quantitative/pretest–posttest design	*n* = 124	e‐Service Learning PYD	18–25 years (*M*_age_ = 20.6 years)	2019–2020	Hong Kong	Catalano 15 PYD construct—Chinese version	This study evaluated the effect of online service‐learning on Positive Youth Development (PYD) in “Project WeCan” during COVID‐19. A pre–post‐test analysis involving 124 university students explored the shift to online learning and service implementation. Results show significant enhancements in certain PYD dimensions, such as social and emotional competence, character strengths, self‐leadership, caring disposition, and service leadership beliefs and values. Overall, the study demonstrated positive shifts in PYD and life satisfaction highlighting the value of service learning in challenging times
14.	Lin and Shek (2021)	Quantitative/pretest–posttest design	Time 1 (T2): *n* = 86 Time 2 (T1): *n* = 130	e‐service learning	T1 Age = 19.92 years T2 Age = *M*_age_ = 19.68 years	2017–2018 2019–2020	Hong Kong, Chengdu, Xi'an, China	Catalano 15 PYD construct—Chinese version	This study evaluated the impact of virtual versus face‐to‐face (FTF) service learning on university students during the COVID‐19 pandemic. The analysis found that both modes were equally effective in enhancing PYD and service leadership. However, a greater positive change in clear and positive identity was noted in students in the virtual modality of service learning
15.	Lin et al. (2022)	Quantitative/Pretest‐post test design	*n* = 166	e‐service leadership learning	*M*_age_ = 20.27 years *M*_age_ = 19.23 years	2021	Chengdu, Xi'an, China	Catalano 15 PYD construct—Chinese version	This study evaluated online service learning (SL) projects in China during the COVID‐19 pandemic. Using a pretest–posttest approach, revealed significant improvements in students' Positive Youth Development (PYD), service leadership, and life satisfaction. The largest changes were observed in competence (cognitive, social, and behavioral) and spirituality Cross‐lagged modeling indicated that students with higher initial PYD showed increased service leadership and life satisfaction, while the initial service leadership did not predict postproject PYD qualities
16.	Marciniak et al. (2022)	Quantitative	*n* = 1362	Relationship between well‐being and civic engagement during the COVID‐19 pandemic Eudaimonic approach.	18–25+ years (*M*_age_ = 22.2 years) Mixed sampling	May to July 2021	(Poland, *n* = 596, Croatia, *n* = 386, and Lithuania, *n* = 379)	Eudaimonic well‐being Civic engagement	This research examined the link between well‐being and civic engagement during the COVID‐19 pandemic among students in Poland, Lithuania, and Croatia. Involving 1362 academic students, the study analyzed mean rank differences in civic engagement based on psychological well‐being levels. Results indicated that higher psychological well‐being correlated with increased civic engagement, particularly in positive relations, personal growth, autonomy, and self‐acceptance dimensions. The findings suggest promoting targeted actions for personal development and relationships during crises like pandemics, emphasizing the globalized nature of student experiences
17.	Miconi et al. (2021)	Mixed method/focus groups and survey	*n* = 31	PYD Community‐based research	14–20 years (M_age_ = 16.71 years) Convenience Sampling	August 2020	Group of Egyptian and Roma minority youth in Albania	Developmental assets	This mixed‐method study explores the accessibility of developmental assets among Egyptian and Roma minority youth in Albania during the COVID‐19 pandemic. Six focus groups were conducted in August 2020 with Egyptian (*n* = 16) and Roma (*n* = 15) adolescents. In addition, adolescents rated how much they experienced each developmental asset. Descriptive and thematic analyses highlighted: (1) low developmental assets and barriers to accessing resources, (2) mental health concerns and coping strategies, and (3) the role of proximal contexts of life, and (4) experiences within the society in terms of discrimination, integration, and contribution to society. Inter‐sectoral community‐based interventions are urgently needed to mitigate the impact of the pandemic on minority youth
18.	Mutch and Estellés (2021)	Qualitative/Participatory Research	*n* = 30	Youth participation in acts and actions of citizenship	16+ year‐old children who were identified by their teachers as being engaged in citizen activities	–	Auckland, New Zealand	Youth citizenship, action & act	Young people engaged in social contributions ranging from discussion of adherence to lockdown measures to take on caring responsibilities for others; negotiating rules and roles (identify formation); enacting individual's own rights and strategies to cope with lockdown; transgressing boundaries or purposive acts to change the status quo of discrimination and social injustice. Need to recognize young people as sites of potential that have to be supported and nurtured by the education system
19.	Owens et al. (2022)	Case study	*n* = 8	Virtual social media use to address social inequity in the school context	11–12‐graders with special educational needs	No time given, but during Covid‐19	USA	Critical service learning/transformative social learning	This case study is guided by a critical service learning (CSL) framework and illustrates the role of school social workers in empowering students through transformative social and emotional learning (TSEL) and youth‐led participatory action research (YPAR). By fostering critical thinking and advocacy skills among students, an integrative approach can enable them to address equity issues in their schools and communities. Students also launched a culturally sensitive media campaign to reach their families and communities. The authors illustrate students' strengths and assets
20.	Sewell (2021)	Quantitative	*n* = 262	University wide volunteering initiative—participation was predicted by individual factors	Two groups *M*_age_ = 21.3 years *M*_age_ = 20.16 years	Jun 2020–December 2020	Illinois, USA	Volunteering	This longitudinal study investigated the social, emotional, and behavioral (SEB) skills and motivations of 262 youth volunteers in a COVID‐19 university initiative. Findings revealed higher perspective‐taking, abstract thinking, and capacity for consistency among volunteers. Those meeting program targets showed better task management and stress regulation. Interestingly, volunteering wasn't linked to altruistic or context‐specific motivations, underscoring the importance of SEB skills in civic engagement during the pandemic
21.	Shek et al. (2022)	Quantitative/pretest–posttest design	*n* = 460	e‐service learning PYD	University students	2020–2021	Chengdu, Xi'an, Hangzhou, China	Catalano 15 PYD construct—Chinese version	This study examines the impact of electronic service‐learning (e‐SL) projects on Positive Youth Development (PYD). Using a combination of pretest‐posttest scores and subjective evaluations from university students and other stakeholders, the study found that university students, as service providers, exhibited enhanced PYD attributes, leadership qualities, and life satisfaction. These improvements were also reflected in their positive perceptions of the learning experience and benefits to themselves and service recipients
22.	Soler‐i‐Martí et al. (2020)	Quantitative (Twitter and Content analysis)	Analysis of Twitter data, youth activism, protests, and demonstrations	Case study of Friday‐for‐Future Movement in Barcelona, Spain: on‐ and off‐line youth activism	–	February 2019–June 2020	Spain	Social movements, online–offline activism	This study examines connections between online and offline activism, leveraging the unique context of the COVID‐19 lockdown and increased digital engagement. Focused on the Barcelona branch of Fridays For Future, the analysis, based on Twitter data, youth protests, and activism, reveals a symbiotic relationship between online and offline actions. During the lockdown, there was a surge in tweets but a decline in interactions, indicating a nuanced impact on social networks
23.	Son and Berdychevsky (2022)	Qualitative/Constructivist Grounded Theory	*n* = 21	Study of Community‐Based Sport and Recreation Programs using purposive snowball sampling	14–17 year‐olds, Practitioners and volunteers aged 18–50 years	–	Chicago, USA	Sports and recreation programs	The study employed a grounded theory approach involving interviews with youths and practitioners to explore the impacts of COVID‐19 on sports and recreation programs. The results indicate that the transition from indoor to outdoor activities and the shift toward volunteer‐driven initiatives, like food distribution, not only changed the focus from leisure to service but also offered unexpected opportunities for developmental growth and enhanced civic responsibility
24.	Song and Hur (2022)	Mixed‐method (case study– quantitative + qualitative)	*n* = 15	Youth leadership	Grade 9th to 12th	–	USA (Korean American adolescents)	Developmental Assets	This community‐based participatory action research program study investigates changes in the leadership development of Korean‐American adolescents during the COVID‐19 pandemic. The findings reveal that the program effectively fostered positive youth development by enhancing participants' communication skills and broadening their perspectives on leadership while encouraging youth to become agents of change in their communities. Additionally, the study highlights the impact of cultural identity on perceived leadership development, noting differences among students who more strongly identify with either Korean or American ethnicities
25.	Wati et al. (2021)	Normative Legal research using secondary data	–	Youth participation	Youth (No age criteria defined)	–	Indonesia	Youth Participation, Public legal awareness	The study examines the role of youth in Indonesia's public health efforts during the pandemic. It emphasizes their contribution to combating vaccine hesitancy, disseminating accurate information, and assisting in mass vaccination programs. Additionally, youth support vulnerable communities during lockdowns with essential services, and the paper argues that youth's active participation is key to the success of health policies and practices during the pandemic
26.	Wilf and Wray‐Lake (2021)	Qualitative/Constant Comparative Analysis	*n* = 20	Youth online civic engagement	16–21 years marginalized youth Purposive sampling	March–September 2020	USA	Online civic engagement, critical consciousness	This study explores online youth civic engagement, focusing on those with historically marginalized identities based on race, ethnicity, immigration status, and religion. The study identifies three forms of online youth civic engagement: Restoring, Building Community, and Taking Collective Action, demonstrating how social media serves as a platform for resisting oppression and injustice, especially for marginalized youth
27.	Yazdani et al. (2022)	Quantitative. Survey data from US college students	*n* = 707	Motivations underlying civic/political engagement	18–22 years (*M*_age_ = 20 years)	April 2020	USA	Communal orientation, civic engagement	This study investigates the impact of the COVID‐19 pandemic on civic engagement among college students. Findings reveal that 70.4% of participants engaged in civic activities, primarily online. Differences in engagement were observed based on socio‐political perspectives, communal orientation, and intended presidential vote, highlighting varied motivations for pandemic‐related civic involvement. The study emphasizes the importance of considering socio‐political factors in understanding and addressing emerging adults' civic engagement during crises
28.	Zhu et al. (2021)	Quantitative/pretest–posttest design	*n* = 228	Online Service leadership qualities, well‐being	*M*_age_ = 19.97 years	2019–2020	Hong Kong	Catalano 15 PYD construct—Chinese version	This study investigated the impact of an online service leadership course, both in asynchronous and synchronous modes, on university students' development during the COVID‐19 pandemic. With 228 students participating in pretest–posttest evaluations, the asynchronous mode was slightly more effective in promoting students' attitudinal change, though the overall effectiveness of both modes was similar. Positive changes were observed in service leadership qualities, life satisfaction, and PYD attributes. Students also reported positive experiences and satisfaction with the course's design and the quality of teaching
**Reports**									
1.	United nations (2021)	Report	500 young human rights defenders, peacebuilders, and community organizers across 96 countries.	How has COVID‐19 affected activism in civic space?Factors contributing to shrinking civic space for youth, identify information gaps and offer recommendations for youth protection	18–29 years	August to November 2020	Africa, Asia‐Pacific, Latin America & the Caribbean, Eastern & Western European countries	Youth protection framework ensures the safe exercise of human rights in the context of civic space	The report based on a survey, online group consultations, and interviews addressed issues of sociocultural barriers and intergenerational hostility; financial challenges and limited employment opportunities, political threats and pressures; legal barriers, and digital (online harassment, censorship, and surveillance) as well as physical (including torture, detention or murder) threats experienced by young activists. The report also identified concerns about specific subgroups of vulnerable youth, such as migrants, refugees, LGBTQT communities, or those with disabilities. The findings suggest that the pandemic and lockdown measures interrupted or slowed down civic activities and engagement. Some young people also reported that the pandemic was used as a justification for the repression of youth‐led protests Identified the lack of dedicated mechanisms, institutions, or structures to provide open, safe, and inclusive spaces for young activists to come together to report and discuss the challenges they are facing and to trigger accountable counter‐measures as a common problem that needs to be addressed by national, international and inter‐governmental organizations
2.	OECD (2020)	Policy brief outlining measures that governments can utilize for inclusive recovery	Survey findings from 90 youth organizations from 48 countries	The brief examines the impact of the crisis on young people and across different age cohorts, as well as its implications for intergenerational solidarity and justice	15–24 years	April 7–20, 2020	48 countries	Youth participation & justice	Based on survey findings this policy brief specifies the need for assessment of the immediate, medium, and long‐term effects of the Covid‐19 pandemic on young people and vulnerable groups. Key issues are mental health, disrupted education, unemployment, poverty, and income, as well as racism and widespread disinformation. Moreover, the report highlights the role of young people as catalysts of inclusive and resilient societies in crisis response, recovery, and in preparation for future crises Practical measures governments can take to design inclusive and fair recovery measures include: The use of youth and intergenerational lens in crisis response and recovery; Collaboration with youth stakeholders by engaging in actionable programs using existing platforms and national youth strategies; Collaboration with national statistics offices and research institutes to strengthen knowledge base on the impact of Covid‐19 by age group and other demographics to inform decision‐making; The use of impact assessments and institutions in anticipating the distributional effects of rule‐making and the allocation of public resources across age groups; Promotion of age diversity in public consultations and state institutions and to inform decision‐making with regards to various age groups; Maximizing young people's mobilization in crisis mitigation through platforms, tools, and mechanisms to build society/community resilience against future shocks and disasters; Aligning short‐term emergency responses with investments into long‐term economic, social, and environmental purpose to secure the welfare of future generations; Providing targeted policies and services for the most vulnerable populations (e.g., NEET, young migrants, homeless youth, children, adolescents, and young women at risk of domestic violence)
3.	United Nations (2022)	Report	130 UN country teams	Meta‐review of studies commissioned by the Office of the Secretary‐General's Envoy on Youth (OSGEY)	18–29 years	August 2020–April 2021	Global, UN country teams	Youth‐centred, human rights‐based approach to COVID‐19 recovery	This meta review reports on programs or activities implemented to address the impact of the COVID‐19 pandemic on young people highlighting the activities of different young leaders and youth organizations from 66 countries and territories. A total of 130 stories of young people and youth organizations were shared between March and August 2020. Several articles focused on marginalized young people, including young people with disabilities, young LGBTIQ+ people, and young refugees Good practices to support young people and ensure the full implementation of their rights during and after the pandemic include the promotion of (1) accountability in working with and for young people; (2) interagency collaboration and collective ownership; (3) decent work for young people; (4) young people's right to meaningfully participate in decision‐making; and (5) ensuring safe online spaces by promoting accountability and awareness about risks related to online youth participation
4.	United Nations Development Programme (2021)	Report	13	Digital youth activism and civic engagement	130 young civic actors, experts, and practitioners from Europe, Central Asia	August 2020—January 2021	Europe, Central Asia	Youth empowerment & capacity building	The rapid analysis based on responses of civic actors from Europe and Central Asia revealed that COVID‐19 had a strong impact on the civic activism of youth. Respondents reported that the digital realm positively enabled direct engagement and improved outreach. Potential barriers to engagement included the lack of digital skills, lack of internet access, and lack of funding supporting digital activism, as well as intergenerational gaps as indicated by the inability to fully interact with policymakers via online platforms. Online and offline activism were viewed as complementing each other

### How did the COVID‐19 pandemic affect the civic engagement of young people?

Our thematic analysis revealed both negative as well as positive outcomes of the COVID‐19 pandemic on young people's lives. As can be seen in Table 2, a number of studies addressed multiple aspects, including both positive and negative ones.

**TABLE 2:** How did the COVID‐19 pandemic affect the civic engagement of young people?

Theme	Subtheme	References
Negative	Limited access to community resources; exacerbated social stratification in civic participation	Kvieskienė et al. (2021), Miconi et al. (2021), OECD (2020), Son and Berdychevsky (2022), Sewell (2021)
	Digital divide; misinformation; declining interest in online activities	Fine et al. (2021), OECD (2020), Kwan (2022b), Soler‐i‐Martí et al. (2020), UNDP (2021), United Nations (2021)
	Distrust in government and disengagement	Chauke (2022)
	Perception of social inequalities: discrimination, financial hardship, unemployment, etc	Chauke (2020, 2022), Fine et al. (2021), Kvieskienė et al. (2021), Kwan (2022a, 2022b), Maker Castro et al. (2022), Miconi et al. (2021), OECD (2020), UNDP (2021), United Nations (2021), Wilf and Wray‐Lake (2021), Yazdani et al. (2022)
	Perception of health risks and experience of loss (including death, family separation, emotional distress, and uncertainty)	Fine et al. (2021), Ingoglia et al. (2022), Kvieskienė et al. (2021), Marciniak et al. (2022), OECD (2020), United Nations (2021)
Positive	Increasing online activism	Fine et al. (2021), Jiang and Gu (2022), Kwan (2022a, 2022b), United Nations (2021), Yazdani et al. (2022), Wilf and Wray‐Lake (2021)
	Innovations in civic engagement programs; service learning	Arnold (2020), Chai et al. (2022), Leung et al. (2021), Lin et al. (2022), Lin and Shek (2021), Owens et al. (2022), Shek et al. (2022), Son and Berdychevsky (2022), Zhu et al. (2021)
	Volunteering: civic competence and responsibility, empowerment; service leadership	Branquinho et al. (2022), Chauke (2020), Ingoglia et al. (2022), Kwan (2022a), Marciniak et al. (2022), Son and Berdychevsky (2022), Sewell (2021), Wati et al. (2021), Yazdani et al. (2022)
	Critical consciousness: perception of social inequality as a catalyst for civic engagement; community building and taking collective action	Fine et al. (2021), Miconi et al. (2021), Kwan (2022b), Miconi et al. (2021), Wilf and Wray‐Lake (2021)

#### Negative outcomes

The lockdown measures led to limited access to community resources, such as safe spaces and support networks (Kvieskienė et al., 2021; Miconi et al., 2021; OECD, 2020; Sewell, 2021; Son & Berdychevsky, 2022) as well as restrictions regarding offline civic activism, with many of the ongoing civic engagement activities being interrupted, slowed down, or having to move online (United Nations, 2021; United Nations Development Programme, 2021; Wilf & Wray‐Lake, 2021). However, not all young people have equal access to online resources which can lead to unequal opportunities for participation in digital civic engagement. A report commissioned by the United Nations based on surveys, interviews, and consultation with young civic actors, experts, and practitioners in the regions of Europe and Central Asia highlights that the majority of respondents felt that the pandemic had aggravated the digital divide and exclusion of youth groups (65% of survey respondents, United Nations Development Programme, 2021). Young people in less privileged circumstances (including those from low‐income backgrounds, rural areas, or with limited technological resources) generally encountered more difficulties in accessing online resources (Kwan, 2022b; OECD, 2020; United Nations, 2022). Thus, while digital activism became more prevalent, some youth groups experienced greater exclusion due to lacking access to necessary digital tools and platforms, suggesting a digital divide.

There is also evidence to suggest declining interest in online‐only activities, for example in a study on young climate activists in Spain (Soler‐i‐Martí et al., 2020) and potential ZOOM burnout, or fatigue with online activities and video‐conferencing (Dacillo et al., 2022). Notably, the growing use of social media also increased the risk of misinformation, undermining trust in social institutions and others (Fine et al., 2021), although there are variations in response across countries, with trust increasing, especially in non‐OECD countries (OECD, 2020). Yet, inconsistent government actions during the pandemic were associated with distrust and political disengagement among youth in South Africa (Chauke, 2022).

There is also evidence to suggest that minority youth characterized by their color, ethnicity, migration status, religion, or sexual identification were exposed to increased levels of discrimination in their living contexts (Fine et al., 2021; Song & Hur, 2022; Wilf & Wray‐Lake, 2021). Some studies reported an increased perception of social needs, injustice, and discrimination among young people (Maker Castro et al., 2022) as well as increased levels of socio‐economic hardship, such as youth unemployment or financial worries (Kvieskienė et al., 2021; Miconi et al., 2021). More generally, a changing economic climate aggravated financial challenges (Chauke, 2020, 2022), but also reduced available funding for supporting safe spaces for young people to meet and engage (United Nations, 2021). In addition, issues related to mental health, disruptions to education, increased stress due to income loss, family well‐being, and worries about life and survival made it difficult for many to concentrate on activism (OECD, 2020; United Nations, 2021). Similar findings were reported in a study on rural Eastern European populations of young people at risk of economic disengagement (Kvieskienė et al., 2021), which argued that the deficit of social interaction, family‐related tensions, problems caused by isolation at home, increased level of anxiety, and mental health problems all contributed to exclusion from society as reflected in the rise of unemployment among young people, in particular among the less educated and socially disadvantaged.

#### Positive outcomes

Regarding potential positive outcomes there is evidence of increased online activism (Fine et al., 2021; Jiang & Gu, 2022; Kwan, 2022a, 2022b; United Nations, 2021; Wilf & Wray‐Lake, 2021; Yazdani et al., 2022) as well as an increasing importance of online efforts (United Nations Development Programme, 2021). Several young activists and community mobilizers stated that their work has become easier, cheaper, faster, and more efficient through the shift to digital technologies and social media (United Nations, 2021). There was also evidence of innovations in civic engagement. For example, evidence from China suggests that online service learning provided during the pandemic can support service leadership and delivery and in turn also well‐being of students (Chai et al., 2022; Leung et al., 2021; Lin et al., 2022; Lin & Shek, 2021; Shek et al., 2022; Zhu et al., 2021). The e‐learning program these studies refer to was developed as a business‐university‐community partnership with the aim to provide disadvantaged local secondary school students with learning opportunities to empower and prepare them for their future education or career. In addition, there is evidence of online activities focused on educating oneself and others about safe living during the pandemic (Jiang & Gu, 2022; Kwan, 2022a, 2022b; OECD, 2020; Pavarini et al., 2020; United Nations, 2021, 2022). A legal study from Indonesia suggests that governments have to recognize the need and value of voluntary youth participation during times of crisis and engage young people in efforts to inform and educate the public about the benefits of public health measures such as the mass vaccination program—which can involve online as well as in‐person engagement (Wati et al., 2021). There is evidence of pandemic‐specific off‐line youth volunteering, such as providing help to others in need such as organizing shopping and delivery services (Branquinho et al., 2022; Kwan, 2022a; Marciniak et al., 2022; Sewell, 2021; Son & Berdychevsky, 2022). Evidence from South Africa suggests that young people developed new hands‐on initiatives of support, such as engaging in social entrepreneurship, distributing food as well as hand sanitizers, masks, and other protective equipment to vulnerable households as well as using digital technology to enhance youth volunteerism and promote healthy lifestyles—all without any government support (Chauke, 2020). Youth involvement in civic activities also helps in empowering youth to identify and respond to community needs, become empathetic, and develop critical thinking skills and a sense of agency (Owens et al., 2022) that may result in developing a civic orientation to continue this work in future (Yazdani et al., 2022).

Moreover, there is evidence to suggest that perceptions of social inequality can serve as a catalyst for civic engagement and taking collective action to resist oppression and injustice (Fine et al., 2021; Kwan, 2022a, 2022b; Miconi et al., 2021; Wilf & Wray‐Lake, 2021). For example, evidence from Singapore illustrates that young people's recognition of existing inequalities, which were exacerbated by the pandemic, also brought with it the effective use of social media for online mobilization to enhance political awareness, activism, and volunteering where young people collaborated across interconnected initiatives and thus facilitated community building (Kwan, 2022b) ‐ or mobilized efforts to emphasize the importance of voting as well as political engagement beyond elections to instigate political change (Kwan, 2022a).

### Which dimensions of civic engagement have been addressed?

The reviewed studies and reports reflect ongoing debates regarding the conceptualization and definition of youth civic engagement (Ekman & Amnå, 2012; Shaw et al., 2014), a construct that comprises multiple dimensions. The reviewed studies can be grouped into the broad themes of political and nonpolitical prosocial actions involving voting, activism, community engagement, volunteering, and civic leadership. Notably, there appear to be interlinkages between the different dimensions as several authors have addressed more than one dimension (see Table 3).

**TABLE 3:** Dimensions of civic engagement.

Manifestations	Dimensions	References
Political	Voting, election campaigning	Kwan (2022a), Yazdani et al. (2022)
	Activism; political protest	Fine et al. (2021), Kwan (2022a), Marciniak et al. (2022), Soler‐i‐Martí et al. (2020), Yazdani et al. (2022)
	Engagement with communities and governments (participation in political process, community building)	Kwan (2022a, 2022b), OECD (2020), UNDP (2021), United Nations (2022), Yazdani et al. (2022)
	Critical consciousness (understood more broadly to include perceived discrimination, social inequality, and social justice)	Chauke (2022), Fine et al. (2021), Kwan (2022b), Maker Castro et al. (2022), Mutch and Estellés (2021), OECD (2020), Owens et al. (2022), United Nations (2021), Wilf and Wray‐Lake (2021)
Nonpolitical	COVID‐19‐specific communication (information on education; vaccination; and health)	Chauke (2020), Ingoglia et al. (2022), Jiang and Gu (2022), Mutch and Estellés (2021), Wati et al. (2021)
	COVID‐19‐specific helping behavior (volunteering, community service, and donations)	Branquinho et al. (2022), Chauke (2020), Kwan (2022b), Marciniak et al. (2022), Mutch and Estellés (2021), Sewell (2021), Son and Berdychevsky (2022), Song and Hur (2022), Yazdani et al. (2022)
	COVID‐19 nonspecific service learning, youth leadership	Arnold (2020), Chai et al. (2022), Leung et al. (2021), Lin et al. (2022), Lin and Shek (2021), Shek et al. (2022), Song and Hur (2022), Zhu et al. (2021)

#### Political activities

Political actions include voting or election campaigning during the pandemic (Kwan, 2022a; Yazdani et al., 2022), as well as youth activism and political protest (Marciniak et al., 2022; Yazdani et al., 2022) aiming to raise awareness of existing inequalities and discrimination (Fine et al., 2021), or ecological concerns (Soler‐i‐Martí et al., 2020). In addition, there was evidence of active engagement with communities and governments, involving participation in community building (Kwan, 2022a; OECD, 2020; UNDP, 2021; United Nations, 2021, 2022; Yazdani et al., 2022), including activities driven by perceived injustice aiming to address societal inequalities and discrimination (Chauke, 2022; Fine et al., 2021; Kwan, 2022b; Maker Castro et al., 2022; Mutch & Estellés, 2021; OECD, 2020; Owens et al., 2022; United Nations, 2021; Wilf & Wray‐Lake, 2021).

#### Nonpolitical activities

Nonpolitical activities included COVID‐19‐specific communication and information services (Chauke, 2020, 2022; Jiang & Gu, 2022; Mutch & Estellés, 2021; Wati et al., 2021), as well as COVID‐19‐specific helping behaviors, volunteering and community service (Kwan, 2022b; Marciniak et al., 2022; Mutch & Estellés, 2021; Sewell, 2021; Son & Berdychevsky, 2022; Song & Hur, 2022; Yazdani et al., 2022). These activities aimed to provide help and support to the most vulnerable in society (delivering food, hygiene products, or medication), or to educate oneself and others about the effects of the pandemic and how to stay safe. In addition, there was evidence of COVID‐19 nonspecific service learning and youth leadership programs (Chai et al., 2022; Leung et al., 2021; Lin et al., 2022; Lin & Shek, 2021; Shek et al., 2022; Song & Hur, 2022; Zhu et al., 2021).

Many of the studies considered multiple activities, including both political and nonpolitical actions, suggesting inter‐linkages across multiple dimensions of civic engagement. In addition to the key dimension of civic engagement generally discussed in the literature (i.e., voting, activism, community engagement, volunteering, and civic leadership), there is also evidence of newly emerging COVID‐19‐specific helping behaviors (i.e., delivery of food, hygiene products, or medication to those in need), or provision of information about health risks and how to stay safe during the pandemic—often using online communication channels. According to Ekman and Amnå's typology of civic engagement (Ekman & Amnå, 2012), these newly emerging activities can be identified as collective actions, aimed to improve conditions in the civil sphere.

Moreover, the evidence suggests that engagement in one domain can beget engagement in other domains. For example, young people who engaged in service delivery also increased their leadership skills (Chai et al., 2022; Leung et al., 2021; Lin et al., 2022; Lin & Shek, 2021; Shek et al., 2022; Song & Hur, 2022; Zhu et al., 2021) and those with high levels of critical reflection and motivation were more likely to engage in activism (Kwan, 2022a, 2022b). More generally, there appears to be an overlap between the activities, reflecting multiple needs during times of crisis such as the necessity to meet basic survival needs (i.e., gaining access to food and hygiene products), a growing awareness of social inequalities, and discrimination that were exacerbated by the pandemic, as well as a demand for information and active engagement in the form of volunteering and political action to initiate change (Chauke, 2020; Fine et al., 2021; Kwan, 2022a; OECD, 2020; United Nations, 2021).

Two studies also discussed the intensity or level of civic engagement, identified by the number (Marciniak et al., 2022) or the frequency of engaging in different political and nonpolitical activities (Yazdani et al., 2022), including volunteering, making donations, or participating in socio‐political activities (such as discussing political topics with others or voting). Evidence from a sample of Eastern European countries, including Poland, Lithuania, and Croatia, suggests that almost all respondents declared that they took part in activities such as voting or discussing social or political topics when meeting other people but only about half of the students in the sample engaged in volunteering or making donations (Marciniak et al., 2022). Similarly, a study among US college students found that students engage less in volunteering or activism, while engagement in social media is more frequent (Yazdani et al., 2022).

### What are the key factors and processes that enable young people to engage with their community or society at large during the global COVID‐19 pandemic?

In our thematic analysis, we identified different resources ranging from individual to community‐level or institutional resources and initiatives (Table 4). Existing research conceptualized civic development either as a bottom‐up approach, regarding young people as citizens whose civic capacities are to be nurtured or as targets for top‐down training interventions (Tzankova & Cicognani, 2019). The studies included in this review considered individual resources, comprising individual characteristics, that is, social, emotional, and cognitive competencies of the young people and indicators of agency that steered their civic engagement. These competencies, in turn, can be fostered, initiated, and empowered through community‐ or institution‐led activities. Community‐led activities comprise initiatives that take place at the community level, such as local youth engagement programs that aim to promote civic engagement of young people (Branquinho et al., 2022), youth leadership (Song & Hur, 2022), or are targeted at vulnerable youth (Kvieskienė et al., 2021; Miconi et al., 2021; Son & Berdychevsky, 2022; Song & Hur, 2022). These programs, in turn, can be supported by local resources or government funding (Arnold, 2020; OECD, 2020; United Nations, 2021, 2022), and most studies (and all reports) point to the importance of institutional‐ or community‐led initiatives to support and facilitate youth civic engagement. However, in addition to top‐down approaches, the studies reviewed here also show evidence of bottom‐up, youth‐led initiatives, especially in low‐to‐middle‐income countries (Chauke, 2020, 2022; OECD, 2020; United Nations, 2021). There are thus possible overlaps and interactions between different initiatives although the focus of the individual studies can be on either the individual, the communities, or social institutions and governments.

**TABLE 4:** Key factors and processes that support civic engagement.

Themes	Sub‐themes	References
Individual resources	Agency: youth‐led initiatives	Chauke (2020, 2022), Jiang and Gu (2022), OECD (2020), United Nations (2022), Branquinho et al. (2022)
	Other orientated civic competencies: perspective taking; empathy; social responsibility; and, cooperation	Ingoglia et al. (2022), Leung et al. (2021), Kvieskienė et al. (2021), Marciniak et al. (2022), Sewell (2021), Yazdani et al. (2022)
	Perceived social inequality as a trigger for engagement	Fine et al. (2021), Kwan (2022a, 2022b), Maker Castro et al. (2022), OECD (2020), Soler‐i‐Martí et al. (2020), United Nations (2021), Wilf and Wray‐Lake (2021), Yazdani et al. (2022), Miconi et al. (2021)
Community‐led activities	Engaging young people through volunteering and active citizenship	Branquinho et al. (2022), Son and Berdychevsky (2022)
	Youth programs to promote active citizenship	Branquinho et al. (2022), OECD (2020), Owens et al. (2022), Song and Hur (2022), UNDP (2021), United Nations (2021), Wati et al. (2021)
	Local policies and initiatives to address the needs of marginalized youth	Fine et al. (2021), Kvieskienė et al. (2021), Miconi et al. (2021), Song and Hur (2022)
Institution‐led activities	Youth programs to promote active citizenship	Arnold (2020), Branquinho et al. (2022), OECD (2020), Owens et al. (2022), Song and Hur (2022), UNDP (2021), United Nations (2021), Wati et al. (2021)
	Youth participation programs to promote trust in institutions, track the impact of inequality, and provide need‐based services for youth	OECD (2020), Wati et al. (2021)
	Programs to promote economic and social inclusion	Arnold (2020), Chauke (2020, 2022), Fine et al. (2021), OECD (2020), United Nations (2021, 2022), Song and Hur (2022), Wilf and Wray‐Lake (2021), Kvieskienė et al. (2021)
	E‐service learning programs	Chai et al. (2022), Leung et al. (2021), Lin et al. (2022), Lin and Shek (2021), Shek et al. (2022), Zhu et al. (2021)
	Out‐of‐school programs	Arnold (2020)
	Virtual youth programs to promote arts, sports, and recreation	Son and Berdychevsky (2022)
	Family support	Miconi et al. (2021)

#### Individual‐level resources and youth‐led initiatives

There is ample evidence of youth‐led initiatives and organizations mitigating the disruptions caused by the pandemic (OECD, 2020). For example, evidence of youth‐led activities in China involved the use of digital and social media to disseminate knowledge about how to live with the virus (Jiang & Gu, 2022). Youth‐led groups have created digital and online tools or campaigns to offer information and practical advice to young people on how to protect oneself and others, to deal with mental health problems, stigma, and discrimination, or how to access vital services such as education and employment support or health care (OECD, 2020). Evidence from Singapore illustrates online mobilization efforts to build awareness and promote sustainable activism and volunteering in response to the COVID‐19 pandemic (Kwan, 2022b) and to revitalize democracy not only during but also beyond the 2020 general election and to bring about political change (Kwan, 2022a).

Some studies highlighted the role of social, emotional, and cognitive competencies as covariates or predictors of volunteering (Sewell, 2021) or socio‐political engagement (Marciniak et al., 2022; Yazdani et al., 2022). For example, evidence from the United States (Sewell, 2021) suggests that volunteers had higher levels of perspective‐taking skills, abstract thinking skills, and capacity for consistency, as well as higher levels of task management and higher levels of stress regulation compared to those who did not volunteer (bringing in a minimum number of hours). Evidence from Italy suggests that social responsibility and civic engagement behavior increase adherence to public health guidelines (Ingoglia et al., 2022). Regarding appraisal of government measures (Ingoglia et al., 2022), the study furthermore suggests that civic competence and interdependent self‐construal were related to the sense of community and civic engagement behavior, which, in turn, predicted attitudes toward preventing contagion and adherence to public health guidelines (e.g., wearing masks). More generally, the findings highlight the importance of examining the alignment between personal and collective interests to understand emerging adults' evaluative and attitudinal experiences during a period of crisis, such as the COVID‐19 pandemic.

Other studies highlighted the associations between critical reflections on community needs and persisting inequality with motivation and action (Maker Castro et al., 2022), including on‐ and offline engagement and initiatives (Kwan, 2022a, 2022b). Moreover, youth‐led organizations have forged new partnerships with the government to amplify their voice and inform decision‐making that concerns them. However, as pointed out in the UN report (United Nations, 2022), these activities have been slowed down by the pandemic and are generally affected by the lack of support and funding for establishing safe and open platforms for youth to enable critical exchange and participation.

There is also evidence to suggest an association between well‐being and civic engagement, that is, a study from Eastern European countries showed that high levels of well‐being are associated with civic engagement (Marciniak et al., 2022). In contrast to this, two studies conducted in South Africa (Chauke, 2020, 2022) emphasize that the lack of resources and discontent about the lack of support for youth unemployment or youth poverty can stimulate the civic engagement of young people who are thrown to their own resources to cope with the challenges of the pandemic.

#### Community‐led activities

Community‐led activities are understood as grassroots initiatives organized and implemented by local community groups, nonprofit organizations, or local leaders. The primary characteristic of community‐led activities is that they originate from within the community, reflecting local needs and priorities. Even if these initiatives receive government or state funding, their design, implementation, and management are driven by community entities. They can include community‐based efforts aimed to engage young people in supporting their communities through volunteering (Son & Berdychevsky, 2022) or active citizenship (Branquinho et al., 2022), or local social policies introduced by municipalities to address the needs of young people during the COVID‐19 pandemic (Kvieskienė et al., 2021). Some studies highlighted the role of community‐led activities in addressing the needs of marginalized youth, such as providing support for ethnic minorities (Miconi et al., 2021; Song & Hur, 2022) or young people not in employment, education, or training (NEET) (Kvieskienė et al., 2021). For example, a community‐based participatory action program aimed at Asian American youth to support their community members affected by the COVID‐19 pandemic (Song & Hur, 2022) enabled the young people to better understand the needs of their community members and to design culturally appropriate action plans to help them. Another study investigated preexisting community‐based recreation and sports programs and showed how they could support young people to cope with the pandemic by switching to online provision (video games, eSport) or creating safe spaces for exercise and maximizing social interactions (Son & Berdychevsky, 2022). Additional outcomes of the shift from leisure activity before the pandemic to public service delivery during the pandemic included youth‐driven initiatives to engage in food distribution during the pandemic or cleaning up the local area (Son & Berdychevsky, 2022). On the whole community‐based initiatives during the pandemic affected youth participation in myriad ways shaping their social lives, economic opportunities, and ability to engage in civic life.

#### Institution‐led activities

Institution‐led activities public investments in programs and initiatives to promote active youth citizenship (Arnold, 2020; Branquinho et al., 2022; OECD, 2020; Owens et al., 2022; Song & Hur, 2022; UNDP, 2021; United Nations, 2021; Wati et al., 2021), to promote trust in institutions (OECD, 2020; Wati et al., 2021), and to promote economic and social inclusion (Arnold, 2020; Chauke, 2020, 2022; Fine et al., 2021; Kvieskienė et al., 2021; OECD, 2020; Song & Hur, 2022; United Nations, 2021, 2022; Wilf & Wray‐Lake, 2021). In particular, schools are highlighted to play a key role in promoting and enabling young people to gain relevant skills and competencies to make their voices heard and to instigate change (Owens et al., 2022). Notably, school‐based interventions should adopt culturally relevant practices (Song & Hur, 2022) as well as a community‐based approach to ensure outreach to potentially vulnerable groups and their integration into society (Fine et al., 2021; Song & Hur, 2022; Wilf & Wray‐Lake, 2021). Other studies point to the crucial role of out‐of‐school programs (Arnold, 2020), as well as virtual youth programs promoting the arts, sports, and recreation (Son & Berdychevsky, 2022) as potential routes to social integration.

In addition, evidence from China suggests that e‐service learning within the education context can support service leadership and delivery and, in turn also, well‐being (Chai et al., 2022; Leung et al., 2021; Lin et al., 2022; Lin & Shek, 2021; Shek et al., 2022; Zhu et al., 2021).

The importance of promoting economic inclusion and civic participation is highlighted across policies in Eastern European countries (Kvieskienė et al., 2021), in particular regarding support for rural youth and young women. For some, in particular, for those with fewer resources or those living in low‐to‐middle‐income countries or minority populations, the importance of government investment in basic resources (such as education, employment and health services) is highlighted (Arnold, 2020; Chauke, 2020, 2022; OECD, 2020; United Nations, 2021, 2022) to enable PYD and to empower young people to actively engage in society (United Nations Development Programme, 2021). Other studies also noted the importance of family support for vulnerable youth (Miconi et al., 2021) in relatively disadvantaged circumstances where no other institutional or community‐level support was accessible or available.

A report by the OECD (2020) based on survey findings from 90 youth organizations across 48 countries highlights the role of government responsiveness to the pandemic as a key influence in supporting young people's trust in institutions, in particular, the importance of integrity, fairness and openness (provision of clear and open data) as well as available support for the most vulnerable groups (inclusiveness). Moreover, governments can play a crucial role in mobilizing young volunteers and youth workers by creating national volunteering platforms as well as local volunteer centers calling for help with food distribution and practical help for fragile or elderly neighbors. These efforts can, in turn, create long‐lasting effects and benefits for society and the economy, opening opportunities for engaging young people in decision‐making, co‐creation, and building back better, thereby strengthening readiness for future shocks. However, a study by the Office of the UN Secretary General's Envoy of Youth (United Nations, 2021) of more than 500 young human rights defenders, peacebuilders, and community organizers identified the lack of dedicated mechanisms, institutions, structures, or funding to provide open, safe and inclusive spaces for young activists to come together to report and discuss the challenges they are facing and to trigger accountable counter‐measures as a common problem that needs to be addressed by national, international, and intergovernmental organizations.

## DISCUSSION

This review is based on 28 studies and 4 reports that met our inclusion criteria which addressed different manifestations of civic engagement in different socio‐cultural contexts. Research has shown that due to the lockdown measures introduced during the COVID‐19 pandemic, many ongoing civic engagement activities were interrupted, slowed down, or had to move online as there were fewer opportunities for activities requiring physical presence. On‐ and offline participation tend to reinforce each other, although online participation is assumed to act as a gateway to offline civic and political engagement (Soler‐i‐Martí et al., 2020). With the shift to increased use of online activities on social media, there was, however, also evidence of a digital divide regarding access to information and communication technologies, of misinformation (Fine et al., 2021; OECD, 2020; United Nations Development Programme, 2021), and declining interest in online‐only activities (Soler‐i‐Martí et al., 2020).

Moreover, the pandemic highlighted social inequalities in access to relevant resources and support, including access to food, shelter, security, education, employment, and health support, as well as social media. A number of studies reported that the perceived social inequalities trigged young people to take action and become engaged (Fine et al., 2021; Kwan, 2022a, 2022b; Maker Castro et al., 2022; Miconi et al., 2021; OECD, 2020; Soler‐i‐Martí et al., 2020; United Nations, 2021; Wilf & Wray‐Lake, 2021; Yazdani et al., 2022)—a finding that emphasizes the social embeddedness of both PYD and civic engagement as well as the reciprocal interactions between individual and context. A deeper understanding of the social environment can bring about the motivation and opportunity to become engaged, and to advocate for oneself and for the wider community (Gonzalez et al., 2020; Sherrod et al., 2005; Shields, 2010; Tyler et al., 2020). For instance, there was a shift to online engagement and increasing use of social media to educate oneself, participate in socio‐political events, raise critical reflections on social and structural inequalities, and mobilize activities, such as providing aid and information to individuals in need (Chauke, 2020; OECD, 2020; Son & Berdychevsky, 2022; United Nations, 2021) or organizing protests against the status quo (Kwan, 2022a, 2022b; OECD, 2020; United Nations, 2021; Wilf & Wray‐Lake, 2021). Institutions and societies, in turn, were encouraged to support youth civic engagement, harness youth‐led initiatives, and value their contributions (OECD, 2020; Wati et al., 2021).

The reviewed studies suggest differences in focus depending on the socio‐cultural context in which young people are embedded. Studies based in high‐income countries tend to emphasize the role of institutional and community‐level support to build individual resources and competencies. These societies may have well‐established frameworks for volunteering and civic participation that are supported by government programs, nonprofit organizations, and community initiatives. For instance, there is a strong emphasis on building individual resources and competencies through structured programs and institutional support (Arnold, 2020; Sewell, 2021). Cultural values in these contexts often highlight individualism and personal achievement, which may lead to a focus on personal development and self‐improvement through civic activities. Additionally, the availability of resources and infrastructure can facilitate diverse forms of engagement, including formal volunteering and advocacy work. In contrast, in many low‐to‐middle‐income countries, socio‐cultural needs and demands might steer civic engagement toward collective and community‐oriented approaches. For example, evidence from studies in Asia points to collective forms of problem‐solving (Jiang & Gu, 2022; Wati et al., 2021) and collaborative learning (Chai et al., 2022; Leung et al., 2021; Lin et al., 2022; Lin & Shek, 2021; Shek et al., 2022; Zhu et al., 2021), involving young people in volunteering and providing help and services to others. Cultural values such as collectivism, community solidarity, and interdependence often play a significant role in shaping these forms of engagement (Flanagan, 2013; Lerner et al., 2021). In these contexts, civic activities may be deeply rooted in traditions of mutual aid and community support, where collaboration and shared responsibility are emphasized.

Moreover, a number of studies conducted in low‐to‐middle‐income countries (Chauke, 2020, 2022) as well as studies among disadvantaged populations (Fine et al., 2021; Kvieskienė et al., 2021; Miconi et al., 2021) shift the focus to the lack of government investments and access to even basic resources and highlight the importance of young people's initiatives and competencies as well as their vulnerabilities (including their precarity as well as mental health). One of these studies also highlights the importance of families in providing crucial support where governments fail (Miconi et al., 2021). When discussing manifestations of civic engagement, one thus has to consider the wider socio‐cultural context in which it is embedded, as forms of engagement might differ depending on socio‐cultural needs, resources, and demands. However, across all cultural contexts, young people demonstrated their willingness to be engaged, to participate, and to take an active role in society, to provide help and support to others in times of crisis, and to make their voices heard.

Across and within countries, crucial factors that support civic engagement include individual‐level resources as well as support from community and social institutions. The findings suggest that youth employed their personal sense of agency as reflected in the plethora of youth‐led initiatives addressing multiple issues associated with the pandemic affecting individuals and families, such as addressing hunger and job loss, information campaigns and dispelling myths about the COVID‐19 virus, mental health support, combatting discrimination and stigma (OECD, 2020; United Nations, 2021, 2022). Youth‐led initiatives reflected young people's need and passion for learning and competence development, their efforts to make a contribution by helping others as well as their political engagement and activism to change the status quo, spanning across the divide of political and nonpolitical activities.

The findings suggest reciprocal interactions between individuals, communities, and government institutions and organizations. At the individual level, higher levels of civic engagement are associated with better emotion regulation, greater leadership development, and a sense of empowerment (Ingoglia et al., 2022; Sewell, 2021). For example, personal resources, such as empathy, responsibility, civic‐mindedness, respect, and interdependent sense of self, were positively associated with both a sense of belonging to a community and civic engagement behavior (Ingoglia et al., 2022). These skills enable individuals to develop awareness that they are part of a community (Flanagan, 2013; Sherrod et al., 2005) and their responsibility to contribute to their community's well‐being. Moreover, young people are more willing to engage if they recognize inequalities. Feeling outraged and angry about disadvantage and seeing oneself as capable of collectively redressing disadvantage are critical predictors of active engagement (Chauke, 2020, 2022; Kvieskienė et al., 2021; Kwan, 2022a, 2022b; Miconi et al., 2021; Song & Hur, 2022).

At the community level, opportunities are created for interaction with peers as well as with social institutions, enabling youth to take on new responsibilities. Community‐based or youth‐led initiatives, in turn, are supported as well as utilized by government institutions to impact inclusive policy‐making and long‐term strategies for disaster mitigation. Community organizations and institutions catalyzed the engagement of youth in civic action, actively reaching out and involving young people, and in some countries providing relevant resources to support action on the ground (United Nations, 2021, 2022). In the presence of the pandemic stress, the youths' personal and social resources, as well as the resources of the communities and institutions, are harnessed by governments to address COVID‐19‐related issues. Crucially, there is a need for safe spaces to practice civic competencies without being judged and the adoption of culturally relevant practices to account for the needs of marginalized groups (United Nations, 2021). However, more research is needed about the culturally relevant factors and processes that facilitate civic engagement in different socio‐cultural contexts.

A thought leader piece focusing on institution‐led activities highlights the need for public investment in PYD, such as local out‐of‐school training and summer programs as implemented in the 21st Century Community Learning Centers (21st CCLC) program in the United States, to support future generations (Arnold, 2020). Unfortunately, demand for these programs outweighs availability, and more needs to be done to effectively address the needs of young people, particularly those in relatively disadvantaged settings who are facing systemic and structural barriers in access to relevant resources and support structures (Song & Hur, 2022; United Nations, 2021; Wilf & Wray‐Lake, 2021). Investments in youth, including investments into their education, health, and employment opportunities, are investments into the future, recognizing that youth success is critical for the welfare of a country. Moreover, young people are vital agents for change. Evidence from China (Jiang & Gu, 2022) and Indonesia (Wati et al., 2021) suggests that governments understand the role of youth as a vital resource and were drawing on young people to disseminate knowledge regarding mass vaccination and social distancing across different communities and to educate the public. In low‐to‐middle‐income countries, the evidence suggests that young people could not rely on government support or investments and that they were affected by unemployment, poverty, and lack of resources (Kvieskienė et al., 2021; OECD, 2020). Nonetheless, they came forward and took the initiative to provide help, assistance, and support to the most vulnerable in society (Chauke, 2020; United Nations, 2021, 2022).

Acknowledging the multiple contributions and initiatives of young people to society during times of global crisis, every effort should be made to secure their education, health, employment, and well‐being. Moreover, their voices should be heard, and their initiatives aiming to improve their lives and that of others should be recognized. We also note that in some circumstances the engagement of young people might run counter to or even challenge government interventions. For example, some of the reviewed studies touched upon defiance or resistance against government restrictions or governmental interventions (Chauke, 2022; Fine et al., 2021; Wati et al., 2021; Yazdani et al., 2022), although this defiance was not the focus of these studies. Future studies will have to explore possible conflicts and how these are addressed in more detail. The takeaway message from the COVID‐19 experience is that when facing delayed or regressive governmental responses, youth and community organizations in times of need should develop parallel structures and countervailing tactics to foster well‐being. There is a need to scale up solutions co‐produced by the youth‐community‐government interface. This can result in facilitating networking and transformative recovery plans that may emerge rooted in grassroots creativity and social justice.

### Limitations

Several limitations need to be considered when interpreting the findings. The studies were highly diverse in terms of design, setting, timing in relation to the pandemic, population, and measures, which reflected our aim to capture a wide range of evidence. The reviewed studies were of mixed quality, as many studies included nonprobability samples recruited from education or community settings, and their representativeness was poorly described. Important aspects of study design, sample characteristics, and findings were not comprehensively described or even missing, which may reflect the opportunistic nature of much of the research we found. Many of the studies reviewed are cross‐sectional, providing a snapshot of civic engagement during the pandemic. There is limited longitudinal data available to assess the long‐term impact of the pandemic on youth civic engagement. Moreover, different cultural and societal settings and levels of restriction are likely to be contributing factors that are not fully considered here. For instance, while some studies focus on disadvantaged populations or regions, the review does not comprehensively address how factors such as gender, disability, or ethnicity intersect with youth civic engagement during the pandemic. This limits our understanding of how different groups may have experienced the pandemic's impacts on civic participation differently, or how these experiences might vary across different cultural settings. In addition, while some studies highlight the role of government support for youth civic engagement, there is still limited understanding of the reciprocal interactions that can support or hinder youth initiatives. This gap calls for a more nuanced understanding of the role of governmental actions in enabling or constraining civic participation during times of crisis. Furthermore, while many studies focus on the shift to online engagement due to pandemic lockdowns, this leaves out detailed analyses of how offline engagement continued or adapted in regions with limited internet access. The reliance on online engagement as a primary avenue for civic participation overlooks the digital divide, especially in low‐income settings, and may not fully capture the experiences of marginalized youth who lack internet access. Notably, some studies touched upon youth resistance or defiance against government interventions, although this was not a central focus. Future research should explore in greater depth how youth navigated tensions between civic engagement and opposition to government restrictions during the pandemic, as this is an underexplored aspect in the current review.

Despite our efforts to be inclusive, we cannot claim that this review provides a comprehensive or global overview of the impact of COVID‐19 on the civic engagement of young people. The geographic distribution of the reviewed studies leaves gaps across and within whole continents. This shows the limitation of our searches to English‐language studies, the use of major databases, and a specific time frame. Although the research team included researchers from a range of cultural and linguistic contexts, future studies should seek to include evidence published in other languages and actively seek cooperation and collaboration with researchers across diverse cultural contexts. Our review focused on studies in the direct aftermath of the pandemic. The rapidly evolving nature of the evidence base may decrease the usefulness of the findings and the implications offered, and future research needs to extend the focus to a longer time frame, also considering changes in response over time. On the issue of developmental changes, our review could not consider or analyze any distinct developmental periods of civic engagement as most studies did not provide results for separate age groups, presenting findings within broader age ranges (generally 16–30 years).

### Implications

The studies and reports reviewed here show similarities and variations in the manifestations of civic engagement and associated predictors. Adopting a socio‐ecological perspective, and conceptualizing civic engagement as an expression of PYD enabled the consideration of individual and contextual factors, including community‐ and institution‐level influences as potential drivers of civic engagement, as well as the reciprocal interactions between these factors. The reviewed studies provided evidence on both political and nonpolitical activities of civic engagement and presented evidence of new forms of COVID‐19‐specific collective actions, such as engagement in helping behaviors or provision of information aimed at improving conditions in the civil sphere. The findings suggest that youth are active agents that shape the communities and institutions in which they are embedded, which in turn shape their experiences and behaviors. Moreover, the expression of civic engagement varies, depending on the wider socio‐cultural context and its specific opportunities, challenges, and demands. In times of crisis, individual‐level resources come to the fore, as well as the need for social and collective action to address the challenges that exhaust individual capacities. The findings highlight the importance of programs aiming to support youth in navigating an evolving and uncertain landscape while meaningfully articulating personal and civic responsibilities. The COVID‐19 crisis challenged young people but also created opportunities for their civic engagement, fostering a sense of agency in youth while strengthening their ability to critically assess and contribute to rebuilding fractured societal structures. Youth empowerment to act ensures that young people are equipped to participate actively in shaping the future, rather than being passive recipients of societal change.

Moreover, the findings highlight the importance of considering the contextually embedded and dynamic processes underlying an individual's civic engagement and gaining a better understanding of the various predictors and facilitators of civic engagement in diverse populations and communities. Future research has to be more mindful of developmental and cultural variations and the multiple challenges facing young people today, including the experience of poverty, precarity, economic uncertainty, and the lack of security or vital institutional support structures to facilitate active engagement and the thriving of young people in society. While the lack of material resources may not deter young people from responding to crises and engaging in civic action, empowering them with confidence and competence is necessary to promote civic engagement among them. This can be achieved through the collaborative efforts between youth, communities, and the institutions that cater to their needs.

## CONFLICT OF INTEREST STATEMENT

The authors declare no conflict of interest concerning the research, authorship, and/or publication of this article.

## CONSENT FOR PUBLICATION

All authors approved the final version of the manuscript for submission.

## Supporting information


Appendix S1.

## Data Availability

A full table of the reviewed studies and summaries of their findings is provided in Table 1. The PRISMA flow diagram (Figure 1) details the studies that we identified and included in our review.
