# Supplementary material for: The COVID‐19 pandemic and young people's civic engagement: A scoping review
Source: J Res Adolesc. 2024 Dec 1;35(1):e13039. doi: 10.1111/jora.13039 (PMC11758488; doi:10.1111/jora.13039)
Supplement: Supplementary file 1 — Appendix S1. [file JORA-35-0-s001.docx]

**Supplementary Material**

**Supplemental Table 1**

*Search Terms and Results from Database Queries*

| **Database** | **Search Terms** |
| --- | --- |
| **PUBMED**  **(*n*=139)** | ("positive youth development" AND (covid OR coronavirus OR corona OR "COVID-19") AND (civic OR “civic engagement” OR empowerment OR "youth participation")) AND (2020:2023/01[pmclivedate]) |
| **Web of Science**  **(*n*= 6)** | "positive youth development")AND(covid OR coronavirus OR corona OR "COVID-19" ) AND (civic OR “civic engagement” OR empowerment OR "youth participation") (All Fields) |
| **SCOPUS**  **(*n*=141)** | ALL ( "positive youth development" ) AND ALL ( covid OR coronavirus OR corona OR "COVID-19" ) AND ALL ( civic OR “civic engagement” OR empowerment OR "youth participation" ) AND ( PUBYEAR = 2020 OR PUBYEAR = 2021 OR PUBYEAR = 2022 OR ( PUBYEAR = 2023 AND PUBDATETXT ( "January 2023" ) ) ) AND ( LIMIT-TO ( SRCTYPE , "j" ) OR LIMIT-TO ( SRCTYPE , "k" ) ) |
| **EBSCO**^b^  **(*n*=231)** | TX "positive youth development" AND TX ( covid OR coronavirus OR corona OR "COVID-19" ) AND TX ( civic OR “civic engagement” OR empowerment OR "youth participation" ) |
| **Google Scholar^c^ (*n*=200)** | "positive youth development" covid\|corona\|coronavirus civic\|"civic engagement”\|empowerment\|"youth participation" |

Note: ^a^ The search was conducted on PUBMED, Web of Science (WOS), SCOPUS, and EBSCO on January 18, 2023. The search strings presented here have been adjusted to replicate the initial search in databases that utilize specific data delimiters (PUBMED, SCOPUS) or filtering the dates through the database’s interface (EBSCO, WOS); ^b^EBSCO: Using the selected terms, a Boolean search was performed with the "search by proximity" and "equivalent subjects" fields to identify relevant publications published in all of EBSCO’s databases. The search results were then restricted to the publication type "Academic Journals"; ^c^Google Scholar: After searching terms, authors manually filtered selected publications between 2020-2022 (November). The results were sorted by relevance (n = 1730), and the first 200 were extracted, following the recommendation of (Bramer (2019, p.149).

**Reference**

Bramer, W. (2019). *Serving Evidence Syntheses: Improving literature retrieval in systematic reviews*. Erasmus University Rotterdam. hdl.handle.net/1765/120107
